# Supplementary material for: Calculation of Crystal-Solution Dissociation Constants
Source: Biomolecules. 2022 Jan 18;12(2):147. doi: 10.3390/biom12020147 (PMC8961641; doi:10.3390/biom12020147)
Supplement: Supplementary file 1 [file biomolecules-12-00147-s001.zip › biomolecules-1532376-supplementary.pdf]

Supplementary Materials
to
Calculation of Crystal-Solution Dissociation Constants

Sergiy O. Garbuzynskiy [1] and Alexei V. Finkelstein [1,2,3,*]

[1] Institute of Protein Research, Russian Academy of Sciences, 142290 Pushchino, Moscow Region, Russia
[2] Biology Department, Lomonosov Moscow State University, 119192 Moscow, Russia
[3] Biotechnology Department, Lomonosov Moscow State University, 142290 Pushchino, Moscow Region, Russia
* Correspondence: afinkel@vega.protres.ru; Tel.: +7-903-257-6694

Supplementary Calculations

1. Calculation of Sublimation Entropy

The sublimation entropy is calculated [1–2] as

$$-\Delta S_{\text{subl}} \equiv S_{\text{crystal}} - S_{\text{vapor}} = R\ln\left[\frac{\delta x_1\delta x_2\delta x_3}{\hat{V}_{\text{vapor}}\cdot e}\right] + R\ln\left[\frac{\delta\beta_1\delta\beta_2\delta\beta_3}{8\pi^2}\right] + R\sum_{i=1}^{n_{\text{rot}}}\ln\left[\frac{\delta\varphi_i}{2\pi}\right] + R\sum_{j=1}^{n_{\text{vibr}}}\ln\left[\frac{\delta\varphi_j}{n_j\Delta\alpha_j}\right]; \quad \text{(S1)}$$

here, R is the gas constant, and the four terms of Eq. (S1) correspond to the four considered types of movements, see Figure 1 in the main text:

The first term stands for the loss of translational entropy; δx_1, δx_2, δx_3 (measured in Å) are ranges of movements along three translational degrees of freedom in the solid phase (it is reasonable to set all of them equal to δx); here, a molecule movement is limited to a volume of $V_{\text{crystal}} = \delta x_1\delta x_2\delta x_3$; $\hat{V}_{\text{vapor}} = k_B T/P_{\text{sat.vapor}}$ is a volume per molecule in saturated vapor (with pressure $P_{\text{sat.vapor}}$ and temperature T), k_B being the Boltzmann constant (the usage of $\hat{V}_{\text{vapor}}\cdot e$ ·rather than simply $\hat{V}_{\text{vapor}}$ follows from standard statistical physics, as explained in Supporting Information to [2]).

The second term stands for the loss of entropy of rotations of the molecule as a whole; $\delta\beta_1$, $\delta\beta_2$, $\delta\beta_3$ are ranges (in radians) of angles of all three rotations of the molecule in the solid phase; it is reasonable to set $\delta\beta_k = \delta x/A_k$, where A_1, A_2, A_3 (measured in Å) are three maximal radii of the molecule calculated from atomic coordinates (see Supporting Information to [2]).

The third term stands for the loss of entropy of rotations inside the molecule; n_{rot} is the number of free rotations around covalent bonds in the molecule in the free state (these rotations are free due to "low" barriers in the torsional potentials whose heights $\gamma < k_BT/2$ (see [2]); $\delta\varphi_i$ is the range of angles of rotation around bond i restricted by the solid phase; it is reasonable to set $\delta\varphi_i = \delta x/B_i$, where B_i (measured in Å) is the maximal radius of groups rotating around the corresponding covalent bond i (see Supporting Information to [2]).

The fourth term stands for the loss of vibrational entropy; n_{vibr} is the number of "soft vibrations" around covalent bonds. Vibrations around covalent bonds are rotations hindered, even in a free molecule, by high ($\gamma \geq k_BT/2$) barriers of torsional potentials. Some ("rigid") vibrations (corresponding to potentials with $\gamma > 35k_BT$, see [2]) are too small to be additionally hindered by crystals at room temperature; these "rigid" vibrations do not affect the sublimation entropy and are not taken into account here. Other vibrations are "soft": they are restricted by crystals and taken into account; $\delta\varphi_i = \delta x/B_j$ is the range of angles for vibrations around bond j in the solid phase; here, B_j (like B_i) is the maximal radius of the smallest group rotating around the corresponding covalent bond; n_j is the number of energy minima for the torsion, in a free molecule, around bond j; as a rule, $n_j = n_j^0$, where n_j^0 is the multiplicity of the torsional potential, but $n_j < n_j^0$ if some energy minima of the torsional potential are greatly elevated by other non-covalent interactions (like the energy of *cis*-rotamer of the peptide bond); however, no such cases emerged for the molecules considered in this work; $\Delta\alpha_j$ is the range of angles of vibrations around bond j in the free state (see Supporting Information to [2]).

Thus, a decrease in entropy of 1 mole of molecules upon their fixation in a crystal is

$$-\Delta S_{\text{subl}} = R\ln\left[\frac{\delta x^3}{\hat{V}_{\text{vapor}}\cdot e}\right] + R\ln\left[\frac{\delta x^3}{8\pi^2 A_1A_2A_3}\right] + R\sum_{i=1}^{n_{\text{rot}}}\ln\left[\frac{\delta x}{2\pi B_i}\right] + R\sum_{j=1}^{n_{\text{vibr}}}\ln\left[\frac{\delta x}{n_j\Delta\alpha_j B_j}\right], \quad \text{(S2)}$$

and the value of δx for each compound can be calculated as

$$\ln[\delta x] = \frac{\left[\ln\left(\frac{k_BT}{P_{\text{sat.vapor}}}\right) - \frac{\Delta S_{\text{subl}}}{R} + \ln[8\pi^2 eA_1A_2A_3] + \sum_{i=1}^{n_{\text{rot}}}\ln[2\pi B_i] + \sum_{j=1}^{n_{\text{vibr}}}\ln[n_j\Delta\alpha_j B_j]\right]}{6 + n_{\text{rot}} + n_{\text{vibr}}} \quad \text{(S3)}$$

where $-\Delta S_{\text{subl}} = -\Delta H_{\text{subl}}/T$ can be calculated from experimentally measured sublimation enthalpy ΔH_{subl} at a given temperature T (=298 K everywhere in this work); $\Delta\alpha_j$ can be obtained using potentials of rotations around covalent bonds; the values $A_1A_2A_3$ and B_i can be obtained from molecular structures (taken from the Cambridge Structural Database, CSD [3]); the values n_{rot}, n_{vibr}, n_j (and n_j^0) can be obtained from the same structures and torsional potentials.

As for δx, in further calculations, we will need the averaged values $\overline{\delta x}$ = 0.84Å and $\overline{\ln[\delta x]} = -0.21$ (as calculated in the previous work [4]).

2. Calculation of Crystal-to-Vapor Dissociation Constants

Now, the values of $\ln(\hat{V}_{\text{vapor}})$ for all used compounds can be calculated as

$$\ln\hat{V}_{\text{vapor}} = \frac{\Delta S_{\text{subl}}}{R} - \ln[8\pi^2 e \cdot A_1A_2A_3] - \sum_{i=1}^{n_{\text{rot}}} \ln[2\pi B_i] - \sum_{j=1}^{n_{\text{vibr}}} \ln\left[n_j \Delta\alpha_j B_j\right] + (6 + n_{\text{rot}} + n_{\text{vibr}})\overline{\ln[\delta x]}. \quad \text{(S4)}$$

Having $-\Delta S_{\text{subl}} = -\Delta H_{\text{subl}}/T$ (see above), we can exclude ΔS_{subl} (which is not measured directly), and obtain

$$\ln\hat{V}_{\text{vapor}} = \frac{\Delta H_{\text{subl}}}{RT} - \ln[8\pi^2 e \cdot A_1A_2A_3] - \sum_{i=1}^{n_{\text{rot}}} \ln[2\pi B_i] - \sum_{j=1}^{n_{\text{vibr}}} \ln\left[n_j \Delta\alpha_j B_j\right] + (6 + n_{\text{rot}} + n_{\text{vibr}})\overline{\ln[\delta x]}. \quad \text{(S5)}$$

Here, like everywhere above, $\hat{V}_{\text{vapor}}$ is expressed in Å^3. However, the required concentration $\left[C_{\text{vapor}}\right]$ is usually expressed not in 1/Å^3 but in mol/L. Since 1 mole includes 6.02×10^{23} molecules, and 1 liter includes 10^{27}Å^3, the value of mol/L is 1/1660Å^3; therefore, $\log(\left[C_{\text{vapor}}\ (\text{mol/L})\right])$ = log(1660) + log(1/$\hat{V}_{\text{vapor}}$ [Å^{-3}]) ≡ 3.220 + 0.4343×ln(1/$\hat{V}_{\text{vapor}}$ [Å^{-3}]) (here, 3.220 = log(1660) and 0.4343 = log(e)). Thus, we obtain the predictions for

$$\log\left[C_{\text{vapor}}(\text{mol/L})\right] = 3.220 + $$

$$0.4343 \times \left\{-\frac{\Delta H_{\text{subl}}}{RT} + \ln[8\pi^2 e A_1A_2A_3] + \sum_{i=1}^{n_{\text{rot}}} \ln[2\pi B_i] + \sum_{j=1}^{n_{\text{vibr}}} \ln\left[n_j \Delta\alpha_j B_j\right] - (6 + n_{\text{rot}} + n_{\text{vibr}})\overline{\ln[\delta x]}\right\} \quad \text{(S6)}$$

and

$$K_{D,\text{vapor}} = [C_{\text{vapor}}(\text{mol/L})]. \quad \text{(S7)}$$

3. An amplitude and the range of movements in a parabolic well

The entropy of a particle is tightly connected with the amplitude of its thermal movements in the well. It is well known that a molecule in a solid body exists in an approximately parabolic potential well; in one dimension, such potential has (Fig. S1) a form

$$E = const + \frac{\gamma}{2}(r - r_0)^2. \quad \text{(S8)}$$

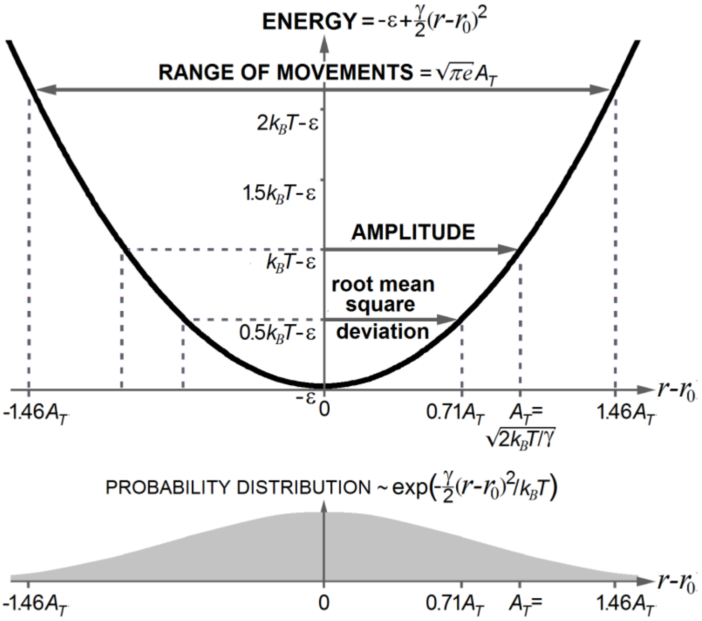

Figure S1. A particle in a parabolic potential well: energy ($-\varepsilon$ is the energy at the bottom of the well), the range of movements, the average amplitude of these movements, and the probability distribution for the particle in the well.

The entropy of a particle in the well is found, from its free energy $F = -k_B T \ln\left[\int_{-\infty}^{\infty} dr \cdot \exp\left(-\frac{\gamma r^2}{2k_B T}\right)\right] = -k_B T \ln\sqrt{2\pi k_B T/\gamma}$, as $S = -\frac{dF}{dT} = k_B T \ln\sqrt{2\pi e k_B T/\gamma}$. Thus, the value $\delta = \sqrt{2\pi e k_B T/\gamma}$ has a sense of the range of thermal movements of the particle in the well. Accordingly, the range of in-crystal thermal movements is greater than the amplitude A_T of these movements by a factor of $\sqrt{\pi e} \approx 2.92$.

Supplementary Tables

Table S1. Thermodynamic experimental data for molecular crystals that melt above 25°C. Extracted from [2,5,6].

№ in this work	№ in whole database [6]	Title	T_{melt}	$\langle \Delta H_{\text{subl}}$	$\pm\delta\langle\frac{\Delta H_{\text{subl}}}{RT}$	ΔU	$\langle \ln k_{\text{H,cc}}$	$\pm\delta\langle \ln k_{\text{H,cc}}\rangle$	$\ln[C_{\text{solution}}]$, experiment
1	7	Trioxane	60.3	56.0	1.1	61.0	9.8	1.9	0.33
2	9	Succinic anhydride	119.0	80.7	0.64	85.7	8.8	2.1	-0.62
3	11	Dimethyl oxalate	54.4	75.2	0.36	80.2	8.9	0.2	-0.29
4	18	*p*-Benzoquinone	115.7	65.2	1.0	70.2	5.8	2.7	-0.99
5	20	2-Nitrophenol	44.8	72.8	0.25	77.8	7.3	0.2	-1.75
6	22	Phenol	40.9	68.9	0.25	73.9	11.1	0.1	-0.06
7	24	*o*-Nitroaniline	71.2	89.0	0.28	94.0	11.3	2.3	-2.04
8	25	*m*-Nitroaniline	114.0	96.8	0.31	101.7	14.96	0.02	-2.06
9	28	Caprolactam	69.2	88.5	0.50	93.5	17.9	2.9	0.83
10	31	1*h*-Benzimidazole	171.0	99.1	1.84	104.1	12.6	2.1	-1.77
11	32	1*h*-Indazole	148.0	91.6	1.22	96.6	10.9	0.4	-2.16
12	33	*p*-Nitrotoluene	51.6	78.0	1.09	84.2	8.34	0.05	-2.49
13	35	*o*-Cresol	29.8	75.4	0.39	81.6	9.95	0.04	-0.62
14	36	*p*-Cresol	35.5	73.7	0.36	79.9	10.15	0.06	-0.70
15	40	2,3-Xylenol	72.8	84.0	0.28	91.4	9.9	0.6	-1.43
16	41	2,5-Xylenol	74.8	84.8	0.11	92.2	9.3	0.8	-1.54
17	42	2,6-Xylenol	45.7	75.4	0.10	82.9	8.3	0.2	-1.31
18	43	3,4-Xylenol	60.8	85.4	0.14	92.8	10.6	0.5	-1.41
19	45	Isoquinoline	26.5	77.1	1.3	82.1	10.2	0.4	-1.46
20	47	Naphthalene	80.2	72.0	0.8	77.0	4.00	0.03	-3.62
21	49	Dibenzofuran	83.0	81.0	1.2	86.0	4.71	0.05	-4.73
22	53	Acenaphthene	93.4	85.0	0.49	90.0	5.00	0.08	-4.60
23	54	Biphenyl	69.0	81.3	0.81	86.3	4.2	0.2	-4.33
24	55	Fluorene	114.8	84.6	1.3	89.6	5.51	0.02	-4.99
25	57	Phenanthrene	99.2	90.3	0.98	95.3	6.36	0.00	-5.22
26	58	trans-Stilbene	123.0	100.5	1.9	105.5	4.3	1.1	-5.79
27	59	Fluoranthene	107.8	100.1	0.73	105.1	7.89	0.03	-5.89
28	60	Pyrene	151.2	101.0	1.30	106.0	7.64	0.02	-6.18

T_{melt} is melting temperature, °C; $\langle \Delta H_{\text{subl}}\rangle$ is the averaged (over literature data, see [5,6], Supporting Information to [2] and references therein) sublimation enthalpy (kJ/mol, at 25°C); $\pm\delta\langle\frac{\Delta H_{\text{subl}}}{RT}\rangle$ is the error of ΔH_{subl} in RT units at T = 298K (25°C); $\Delta U = \Delta H_{\text{subl}} - RT(1-(6+n_{\text{rot}})/2)$ (where n_{rot} is the number of free rotations around covalent bonds in the free molecule) is a change in potential energy (also in kJ/mol); $\langle \ln k_{\text{H,cc}}\rangle = \langle \ln\left(\frac{[C_{\text{solution}}]}{[C_{\text{vapor}}]}\right)\rangle$ is the averaged (over literature data, see [6] and references therein) logarithm of Henry's law constants; $\pm\delta\langle \ln k_{\text{H,cc}}\rangle$ is the error of logarithm of Henry's law constants; $\ln[C_{\text{solution}}]$ is the logarithm of experimentally measured equilibrium concentration (measured in mol/L) in aqueous solution (from literature data, see [6] and references therein).

Table S2. Geometric and energetic parameters concerning rotations of whole molecules and of their parts

№ in this work	Title	Spatial structure	Cambridge Structural Database (CSD) code [3]	n_{rot}
1	Trioxane	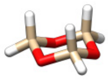	TROXAN11	0
2	Succinic anhydride	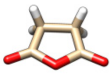	SUCANH13	0
3	Dimethyl oxalate	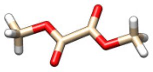	DMEOXA01	0
4	*p*-Benzoquinone	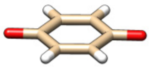	BNZQUI03	0
5	2-Nitrophenol	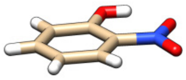	ONITPH	0
6	Phenol	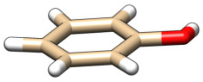	PHENOL03	0
7	*o*-Nitroaniline	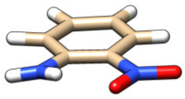	ONITAN	0
8	*m*-Nitroaniline	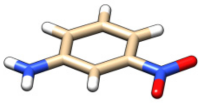	MNIANL03	0
9	Caprolactam	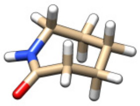	CAPLAC	0
10	1*h*-Benzimidazole	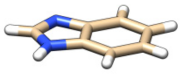	BZDMAZ02	0
11	1*h*-Indazole	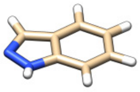	INDAZL	0
12	*p*-Nitrotoluene	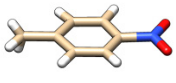	NITOLU	1
13	*o*-Cresol	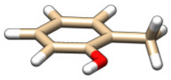	OCRSOL	1
14	*p*-Cresol	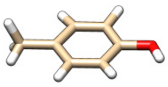	CRESOL01	1

15	2,3-Xylenol	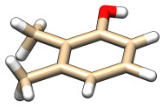	DIMPHE12	2
16	2,5-Xylenol	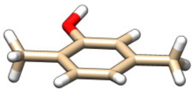	DMPHOL11	2
17	2,6-Xylenol	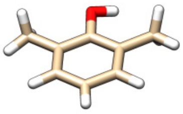	DMEPOL10	2
18	3,4-Xylenol	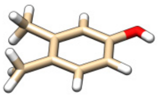	DPHNOL10	2
19	Isoquinoline	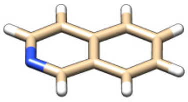	BERXOL	0
20	Naphthalene	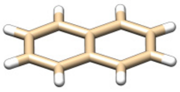	NAPHTA10	0
21	Dibenzofuran	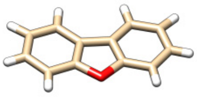	DBZFUR11	0
22	Acenaphthene	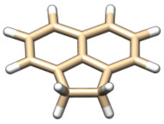	ACENAP03	0
23	Biphenyl	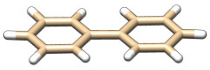	BIPHEN04	0
24	Fluorene	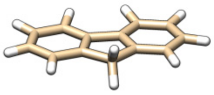	FLUREN01	0
25	Phenanthrene	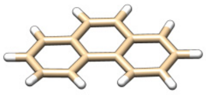	PHENAN08	0
26	trans-Stilbene	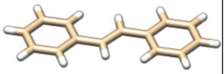	TSTILB10	0
27	Fluoranthene	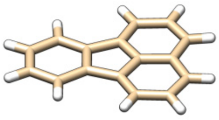	FLUANT02	0
28	Pyrene	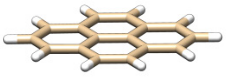	PYRENE02	0

Table S3. Geometric and energy parameters concerning vibrations of parts of the molecules around covalent bonds with "moderately high" barriers (from k_BT to $70\,k_BT$ high). If there are several types of bonds with such barriers in a molecule, each type is listed here separately.

Compound № in this work	First type of bond		Second type of bond		Third type of bond	
	n_{vibr}	γ_j, kcal/mol [7]	n_{vibr}	γ_j, kcal/mol [7]	n_{vibr}	γ_j, kcal/mol [7]
1	0	–	0	–	0	–
2	0	–	0	–	0	–
3	2	0.6	2	1.8	1	10
4	0	–	0	–	0	–
5	1	1.65	1	10	0	–
6	1	1.65	0	–	0	–
7	1	10	1	10	0	–
8	1	10	1	10	0	–
9	0	–	0	–	0	–
10	0	–	0	–	0	–
11	0	–	0	–	0	–
12	1	10	0	–	0	–
13	1	1.65	0	–	0	–
14	1	1.65	0	–	0	–
15	1	1.65	0	–	0	–
16	1	1.65	0	–	0	–
17	1	1.65	0	–	0	–
18	1	1.65	0	–	0	–
19	0	–	0	–	0	–
20	0	–	0	–	0	–
21	0	–	0	–	0	–
22	0	–	0	–	0	–
23	1	20	0	–	0	–
24	0	–	0	–	0	–
25	0	–	0	–	0	–
26	2	10	1	10	0	–
27	0	–	0	–	0	–
28	0	–	0	–	0	–

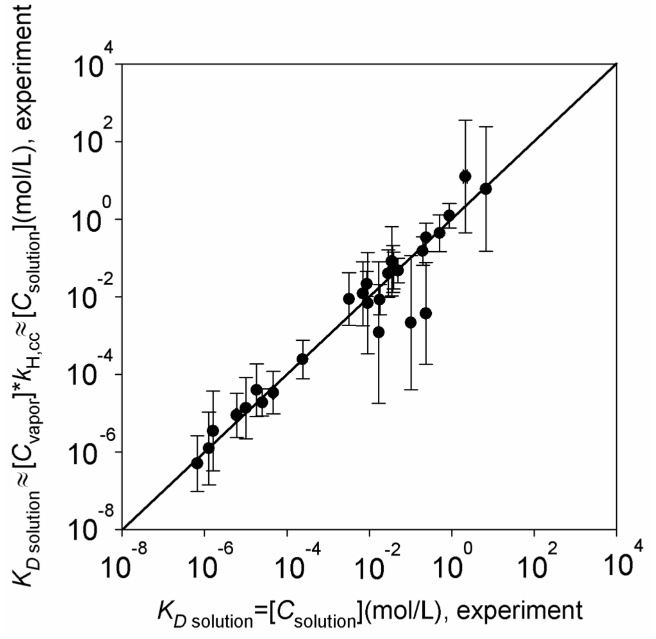

Figure S2. A comparison of the directly experimentally measured $[C_{\text{solution}}]$ with that estimated as a product of two experimental values, $[C_{\text{vapor}}] \times k_{\text{H,cc}}$. Correlation coefficient between these two values is 96%.

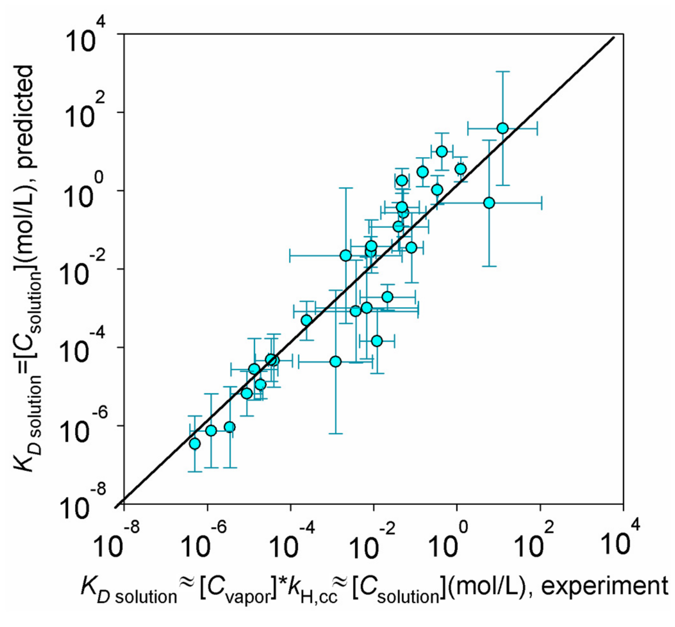

Figure S3. A comparison of the calculated in this work $[C_{\text{solution}}]$, predicted with that estimated as a product of two experimental values, $[C_{\text{vapor}}] \times k_{\text{H,cc}}$. Correlation coefficient between these two values is 93%. A comparison of the calculated in this work $[C_{\text{solution}}]$, predicted with the directly experimentally measured $[C_{\text{solution}}]$ is shown in Figure 3 of the main text.

References

1. Garbuzynskiy, S.O.; Finkelstein, A.V. Calculation of mobility and entropy of the binding of molecules by crystals. *Mol. Biol. Engl. Transl.* **2016**, *50*, 520–529.
2. Garbuzynskiy, S.O.; Finkelstein, A.V. Sublimation entropy and dissociation constants prediction by quantitative evaluation of molecular mobility in crystals. *J. Phys. Chem. Lett.* **2017**, *8*, 2758–2763.
3. Allen, F.H. The Cambridge Structural Database: A quarter of a million crystal structures and rising. *Acta Cryst.* **2002**, *B58*, 380–388.
4. Garbuzynskiy, S.O.; Finkelstein, A.V. Correction to "Sublimation entropy and dissociation constants prediction by quantitative evaluation of molecular mobility in crystals". *J. Phys. Chem. Lett.* **2018**, *9*, 6883.
5. Pereyaslavets, L.B.; Finkelstein, A.V. Development and testing of PFFsol1.1, a new polarizable atomic force field for calculation of molecular interactions in implicit water environment. *J. Phys. Chem. B* **2012**, *116*, 4646–4654.
6. Supplements to "Pereyaslavets, L.B.; Finkelstein, A.V. Development and testing of PFFsol1.1, a new polarizable atomic force field for calculation of molecular interactions in implicit water environment. *J. Phys. Chem. B* **2012**, *116*, 4646–4654.": Supplement A1 <http://phys.protres.ru/resources/FFS/A1en.doc>; Supplement A2 <http://phys.protres.ru/resources/FFS/A2.pdf>, and Additions to A2 <http://phys.protres.ru/resources/FFS/Addition%20to%20A2.pdf>
7. Levitt, M.; Hirshberg, M.; Sharon, R.; Daggett, V. Potential energy function and parameters for simulations of the molecular dynamics of proteins and nucleic acids in solution. *Computer Physics Commun.* **1995**, *91*, 215–231.
